# Inferring occluded projectile motion changes connectivity within a visuo-fronto-parietal network

**DOI:** 10.1007/s00429-024-02815-2

**Published:** 2024-06-25

**Authors:** Gabrielle Aude Zbären, Manu Kapur, Sarah Nadine Meissner, Nicole Wenderoth

**Affiliations:** 1 Neural Control of Movement Lab, Department of Health Science and technology, ETH Zurich, Zurich, Switzerland; 2 Professorship for Learning Sciences and Higher Education, ETH Zurich, Zurich, Switzerland; 3 Future Health Technologies, Singapore-ETH Centre, Campus for Research Excellence And Technological Enterprise (CREATE), Singapore, Singapore

**Keywords:** Physical inference, Visual imagery, fMRI, Functional connectivity, Effective connectivity, Dynamic causal modelling

## Abstract

Anticipating the behaviour of moving objects in the physical environment is essential for a wide range of daily actions. This ability is thought to rely on mental simulations and has been shown to involve frontoparietal and early visual areas. Yet, the connectivity patterns between these regions during intuitive physical inference remain largely unknown. In this study, participants underwent fMRI while performing a task requiring them to infer the parabolic trajectory of an occluded ball falling under Newtonian physics, and a control task. Building on our previous research showing that when solving the physical inference task, early visual areas encode task-specific and perception-like information about the inferred trajectory, the present study aimed to (i) identify regions that are functionally coupled with early visual areas during the physical inference task, and (ii) investigate changes in effective connectivity within this network of regions. We found that early visual areas are functionally connected to a set of parietal and premotor regions when inferring occluded trajectories. Using dynamic causal modelling, we show that predicting occluded trajectories is associated with changes in effective connectivity within a parieto-premotor network, which may drive internally generated early visual activity in a top-down fashion. These findings offer new insights into the interaction between early visual and frontoparietal regions during physical inference, contributing to our understanding of the neural mechanisms underlying the ability to predict physical outcomes.

**Supplementary Information:**

The online version contains supplementary material available at 10.1007/s00429-024-02815-2.

## Introduction

The ability to anticipate the behaviour of objects in the physical environment is crucial for many of our daily actions, such as crossing a busy street or catching a ball. Converging computational and behavioural evidence suggests that this ability relies on an ‘intuitive physics engine’ that predicts future events by running mental simulations (Bates et al. 2015; Battaglia et al. 2013; Gerstenberg et al. 2017, 2021; Hamrick et al. 2016). Intuitive physical inference has consistently been linked to the activation of a frontoparietal network comprising the supramarginal gyrus (SMG), superior parietal lobule (SPL), and dorsal premotor cortex/supplementary motor area (PMd/SMA) (Fischer et al. 2016). These regions have been shown to contain invariant representations of physical properties, providing evidence for a generalised neural intuitive physics engine (Pramod et al. 2022; Schwettmann et al. 2019). Another line of neuroimaging research into intuitive physics provided evidence that early visual areas are also involved in the process. These regions have been shown to contain representations of inferred physical scenarios that resemble those evoked by perception, suggesting that intuitive physical inference may be accompanied by visual simulation of the inferred scenario (Ahuja et al. 2022; Zbären et al. 2023). Interestingly, during explicitly instructed visual imagery, internally-generated visual cortex activity is modulated by frontoparietal areas similar to those involved in physical inference (Dentico et al. 2014; Dijkstra et al. 2017; Ishai et al. 2000; Mechelli 2004), suggesting a potential role of these regions in generating perception-like images when inferring occluded physical scenes. While there is a growing body of evidence supporting the involvement of specific brain areas in intuitive physics, the underlying connectivity patterns between these regions remain largely unknown.

In a recent study, we investigated the neural representations associated with predicting the parabolic trajectory of an occluded ball falling under Newtonian physics (Zbären et al. 2023). We designed an intuitive physical inference task requiring participants to infer the trajectory of an occluded ball falling parabolically. We first established that participants could learn to accurately predict the ball’s trajectory despite the absence of visual input, suggesting that they have successfully built and relied on a mental model of the physical scene to infer the outcomes. We then showed that solving this task activates early visual regions together with a frontoparietal network, and that early visual regions represent task-specific and perception-like information about the inferred trajectory. These results suggest that the outcomes of physical inferences may be represented in form of the perceivable sensory consequences in early visual areas, despite the absence of visual stimulation. Building upon these findings, the aim of the present study was to examine connectivity patterns among brain regions involved in physical inference and whose activity is linked to early visual processing when predicting the trajectory of objects falling parabolically and under occlusion.

First, we aimed to identify regions that may drive or be influenced by early visual activity during physical inference, by conducting a psychophysiological interaction (PPI) analysis. Our findings revealed that all regions consistently involved in intuitive physical inference exhibited increased functional coupling with early visual areas when predicting the parabolic trajectory of occluded objects. Given the absence of task-relevant visual inputs during our physical inference task, the measured neural activity within this network reflects internally generated representations of the physical scene and thus, the information flow between sensory and higher-order areas is unclear. To examine this, we investigated directed connectivity changes associated with physical inference of occluded trajectories in the network of regions revealed by the PPI analysis, using dynamic causal modelling (DCM; Friston et al. 2003). More specifically, we compared various anatomically plausible dynamic causal models to test whether physical inference primarily modulates visual-to-parietal connections, parietal-to-visual connections, or both, and whether it primarily modulates parietal-to-premotor connections, premotor-to-parietal connections, or both.

## Materials and methods

The data used in this manuscript have previously been published in Zbären et al. (2023), which primarily focused on univariate and multivariate pattern analysis of the BOLD signal. Here, we have re-analysed the same data with an emphasis on functional connectivity analysis and network modelling using DCM. The experimental paradigm and pre-processing pipeline are identical to Zbären et al. (2023). We restate the relevant details here for the readers’ convenience.

### Participants

Twenty healthy volunteers participated in the study and four were excluded from the analyses (for detailed exclusion criteria, see Zbären et al. 2023). The final sample consisted of sixteen participants (10 females; 6 males; mean age: 28.31 ± 9.26) with normal or corrected-to-normal vision. The study was approved by the Ethics Committee of the Swiss Federal Institute of Technology (EK 2020-N-31; Zurich, Switzerland) and conducted in accordance with the declaration of Helsinki. All participants provided written informed consent before participation and received monetary compensation upon completion.

### fMRI task

To investigate intuitive physical inference, we designed a task that required participants to predict the fall time and landing location of an occluded ball falling parabolically. Participants were exposed to a dynamic 3D physics environment generated using the Unity3D physics engine (version 2019.2.3; http://unity3d.com). The study consisted of a behavioural training session, followed by an fMRI session happening no more than 7 days later. All participants were naïve to the purpose of the experiment throughout both sessions.

The behavioural session included instruction and training on the physical inference task, with participants receiving written feedback on their performance after each trial. During the fMRI session, participants performed a physical inference task that was nearly identical to the one they had been trained on, but without receiving any feedback on their performance. Throughout the fMRI session, the physical inference task was alternated with a visually matched control task. A cross was displayed at the centre of the screen during both sessions, and participants were instructed to fixate it while performing both the physical inference and control tasks.

**Fig. 1:**
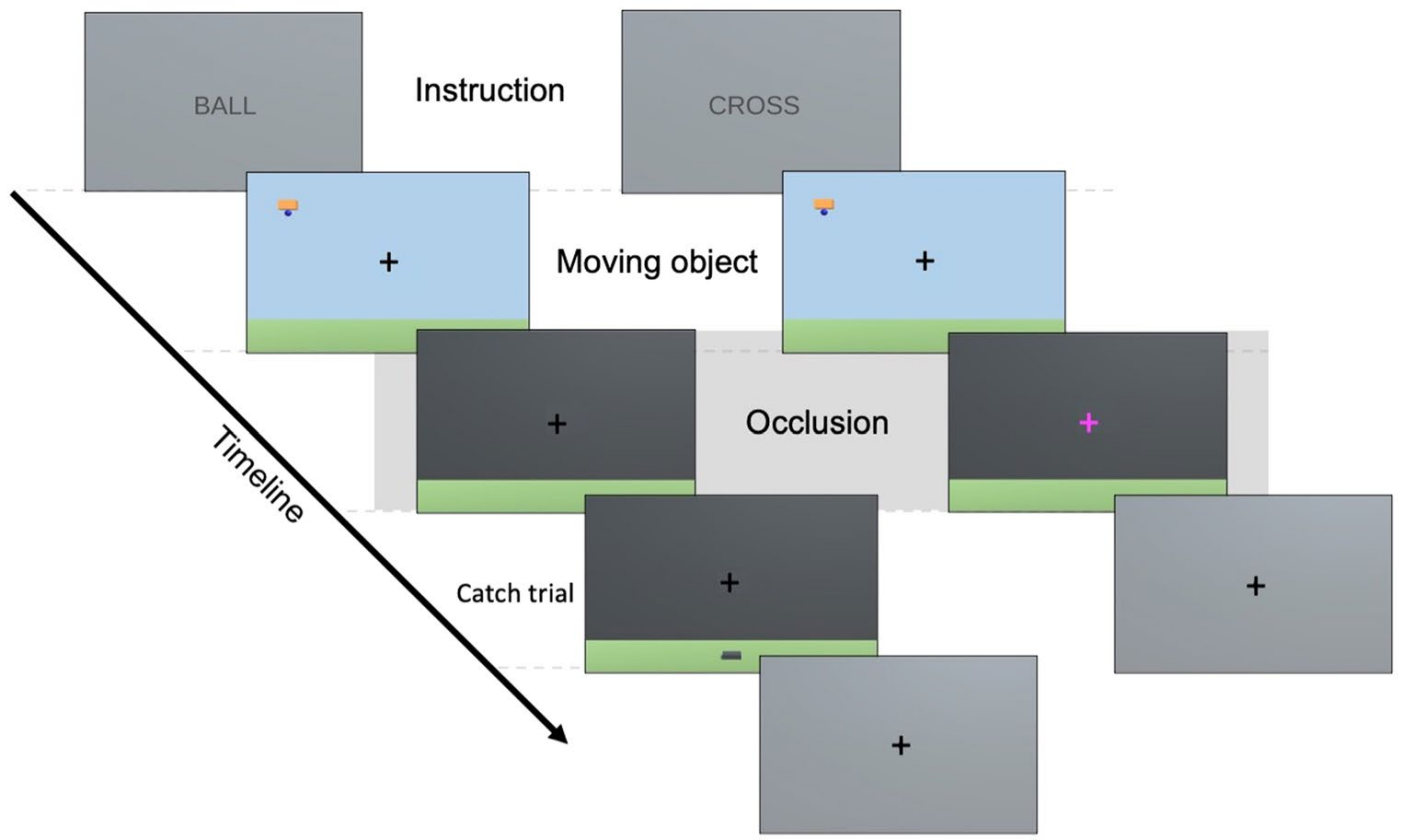
fMRI task. The left and right columns represent the sequence of events in an example physical inference and control trial, respectively. Each block starts with a word cue indicating the task to be performed (Instruction) presented for 3 s. During each trial, participants first view a horizontally moving object with a ball attached, coming from either the left or the right side of the screen (Moving object). The moving object phase lasts between 4.8 and 7.7 s, depending on the velocity of the moving object. Once the object reaches the centre, the ball is released and the screen gets occluded such that neither the moving object nor the falling ball are seen (Occlusion). During the occlusion phase, which lasts 5 s, participants have to press a button to indicate when they think the ball lands in the physical inference condition, and when the colour of the fixation cross changes in the control condition. In some catch trials of the physical inference condition, participants additionally have to indicate where they think the ball lands (Location estimation). They have 8 s to move the basket to the estimated location. Every trial is followed by a 3 s rest period during which a grey screen and fixation cross is displayed

In each trial of the physical inference task, participants were presented with an object moving horizontally either from right to left or from left to right, whose height and velocity varied across trials (Fig. 1). The object carried a ball that was dropped suddenly, at which point the screen was occluded such that neither the object nor the falling ball could be seen. The scene followed Newtonian physics, with the ball entering projectile motion as soon as it started to fall. Subsequently, participants were required to estimate: (i) when the ball would reach the ground (i.e., ‘fall time estimation’), indicated by a button press, and (ii) where the ball would land, indicated by moving a basket on the bottom of the screen to the estimated location. During the fMRI session and in contrast to the behavioural session, participants were not prompted to indicate their location estimation in every trial but only in one catch trial for every six trials. The trials of the control task featured the same visual stimuli but instead of pressing a button to indicate fall time estimation, participants had to press a button as soon as the colour of the fixation cross changed. The timings of the colour changes were randomly drawn from a distribution ranging from the minimum to the maximum true fall times ± 500 ms. Every trial was followed by a 3 s rest period.

The fMRI experiment consisted of 6 runs, each containing the same 18 physical inference and 18 control trials but differently pseudo-randomised. The 18 trials were generated by combining 3 heights and 3 velocities (i.e., [44, 61, 78 m] x [1.3, 1.7, 2.1 m/s]), resulting in trials featuring varying true fall times and locations. Within each run, trials were presented in 3 blocks of 6 physical inference trials, and 3 blocks of 6 control trials. Each block started with a word cue indicating the task to be performed: ‘ball’ (i.e., physical inference task) or ‘cross’ (i.e., control task). The blocks were alternated within each run, with half of the runs starting with the physical inference and the other half with the control condition. One run lasted 10.42 minutes.

### Behavioural and self-rating data

The behavioural data were processed in Matlab (version 9.9; The Mathworks Inc, Natick, MA). To quantify performance, we calculated fall time errors by subtracting the estimated fall times from the true fall times. For each participant, the absolute time error of each trial was computed and then averaged across all trials of the physical inference condition. Additionally, we assessed self-rated vividness. In a post-fMRI debriefing questionnaire, participants were asked whether they ‘imagined the falling ball (i.e., saw it in their mind’s eye) during the experiment’ and if so, to rate the vividness of the image on a visual-analogue scale. The scale ranged from 0, corresponding to ‘No image at all, I only “know” I am thinking of the object’ to 10, corresponding to ‘Perfectly realistic, as vivid as real seeing’.

### fMRI data acquisition and pre-processing

MRI data were acquired on a 3 tesla Philips Ingenia system using a 32-channel head coil. Anatomical images were acquired using a T1-weighted sequence (160 sagittal slices, voxel size = 1 mm3, TR = 8.3, TE = 3.9 ms, flip angle = 8°, matrix size = 240 × 240, FOV = 240 mm (AP) x 240 mm (RL) x 160 mm (FH)). Functional images were acquired using a whole-brain echo-planar imaging (EPI) sequence (40 interleaved transversal slices, TR = 2500, voxel size = 2.75 × 2.75 × 3.3 mm, TE = 35 ms, flip angle = 82°, matrix size = 80 × 78, FOV = 220 mm (AP) x 220 mm (RL) x 132 mm (FH), 250 volumes per run).

fMRI data were pre-processed using FSL version 6.0 (https://fsl.fmrib.ox.ac.uk/fsl/fslwiki). After discarding the first 4 volumes to account for T1 saturation effects, the following pre-processing steps were applied to each run: motion correction using the Motion Correction Linear Image Registration Tool (Jenkinson et al. 2002), brain extraction using the automated Brain Extraction Tool (BET; Smith 2002), spatial smoothing using a Gaussian kernel of 5 mm full-width-at-half-maximum (FWHM), and high-pass filtering using a 100s cut-off as implemented in FSL’s Expert Analysis Tool (FEAT). Each run was additionally inspected for excessive motion and excluded from further analyses if the absolute mean displacement was greater than ~ half the voxel size (i.e., 1.4 mm); two runs (from two different participants) were excluded. Normalisation was performed by aligning functional images to structural ones using boundary-based registration (Greve and Fischl 2009), aligning structural images to the 2 mm Montreal Neurological Institute (MNI-152) standard space using nonlinear registration (FNIRT), and applying the resulting warp fields to the functional images.

### fMRI data analysis

#### Psychophysiological interaction analysis

The PPI analysis was performed using FSL version 6.0. To define the seed region, we used a standard contrast analysis with a general linear model (GLM) based on a double gamma hemodynamic response function (HRF) and its first temporal derivative. The design matrix contained the following two regressors of interest: a ‘physical inference’ regressor modelling the physical inference task, i.e., the period between the start of the occlusion and the button press (indicating the estimated landing time of the ball) minus 500 ms to account for motor preparation, and a ‘control’ regressor modelling the control task, i.e., the period between the start of the occlusion and the colour change. Additionally, there were five regressors of no interest, modelling the periods of the (i) instructions (ball or cross), (ii) horizontally moving object, (iii) button presses (including 500 ms of motor preparation in the physical inference condition, and the time between the colour change and button press in the control condition), (iv) the occlusion period of missed trials in which there were no button presses, and (v) location estimation in the catch trials. Six motion parameters (i.e., rotations and translations along the x, y, and z-axes), as well as white matter (WM) and cerebrospinal fluid (CSF) time-series, were added as nuisance regressors in the GLM. To further reduce motion artifacts, volumes with an absolute mean displacement greater than half the voxel size were scrubbed.

The seed region was created by intersecting an anatomical mask covering V1, V2, and V3 from the Jülich Histological Atlas (Eickhoff et al. 2007), with the group random-effects activation map revealed by the physical inference > control contrast, thresholded at Z > 3.1 and FWE-corrected using a cluster significance level of p_FWE_ < 0.05. The ROI was then transformed to each participant’s native functional space, and its time-course extracted. The first-level design matrix of the PPI analysis comprised the following regressors: (i) the contrast between the occluded phase of the ‘physical inference’ versus ‘control’ condition, convolved with a double gamma HRF (i.e., task regressor), (ii) the time-course of the seed-region (i.e., physiological regressor), (iii) the product of the zero-centred task and de-meaned physiological regressors (i.e., interaction term), (iv) an ‘occlusion’ regressor combining the occluded periods of the ‘physical inference’ and ‘control’ conditions, and (v) the same regressors of no interest and nuisance regressors as described above. The interaction term allows the identification of regions exhibiting task-related covariance with the seed region. Accordingly, an ‘interaction term > rest’ contrast was defined for each participant, and the resulting image entered into a mixed effects higher-level analysis. The group z-statistic images were thresholded at Z > 3.1 and corrected for family-wise-error (FWE) using a cluster significance level of p_FWE_ < 0.05.

To test whether functional connectivity strength is associated with the behavioural and self-rating data, two stepwise multiple linear regression analyses (*p* < .05) were performed: one with the time estimation performance and one with the self-rated vividness used as a dependent variable (see Sect. 2.3). In both regression analyses, the predictors consisted of the mean parameter estimate of each significant cluster revealed by the PPI analysis.

#### Dynamic causal modelling

To investigate causal interactions between the brain regions identified through the PPI analysis, we used dynamic causal modelling (DCM, Friston et al. 2003) implemented in Statistical Parametric Mapping (SPM12, http://www.fil.ion.ucl.ac.uk/spm/). DCM for fMRI is a neurophysiologically plausible modelling scheme that estimates task-related changes in effective connectivity from measured BOLD signals, within a network of preselected brain regions. In DCM, neural activity changes are characterised by the following state-space equation (Eq. 1):

$$\dot{z}=\left(A+\sum _{j=1}^{m}{u}_{j}{B}^{j}\right)z+Cu$$

The state vector $\dot{z}$ represents changes in neural activity over time as a function of the current level of neural activity $z$, the experimental stimuli $u$, and the connectivity parameters $A$, $B$, and $C$. The matrix $A$ specifies the intrinsic or endogenous effective connectivity between and within regions, while the matrix $B$ specifies the changes in effective connectivity due to task-related modulatory inputs ${u}_{j}$. The matrix $C$ represents the direct effects of driving inputs $u$ on a given region. The values of extrinsic connections have units in Hertz (Hz) and represent synaptic rate constants (i.e., connection strengths) while intrinsic (within-region) connections are log-scaling parameters.

##### General linear models

To perform the DCM analysis, the fMRI data pre-processed in FSL was first transformed from native to MNI space, after which two separate first-level GLMs were implemented in SPM: one for time-series extraction and the other for specifying the DCM inputs. The reason for using two separate GLMs was to avoid a rank deficient design matrix that would have resulted from combining the three necessary regressors (i.e., ‘physical inference’, ‘control’, and a combination of both) into a single GLM. Both GLMs included the same five regressors of no interest modelling the periods of the instructions, moving objects, button presses, missed trials, and location estimations (see Sect. 2.5.1). In addition, both GLMs contained the same nuisance regressors consisting of six motion parameters (i.e., rotations and translations along the x, y, and z-axes) and the scrubbing regressors. The GLM used for time-series extraction (GLM1) contained a ‘physical inference’ and a ‘control’ regressor of interest, modelling the occlusion period of the corresponding condition. The GLM used for DCM specification (GLM2) contained the following two regressors of interest: an ‘occlusion’ regressor combining the occlusion periods of the ‘physical inference’ and ‘control’ conditions, and the same ‘physical inference’ regressor as in GLM1. The ‘occlusion’ regressor was used as a driving input, and the ‘physical inference’ regressor as modulatory inputs. As such, the inputs of matrices *A* and *B* were not redundant to each other (see Eq. 1). All task regressors were convolved with a canonical hemodynamic response function (HRF) and its first temporal derivative.

##### Selection of regions of interest and time-series extraction

ROIs were selected based on the results of our PPI analysis and previous research characterising the regions systematically involved in intuitive physics (Fischer et al. 2016; Pramod et al. 2022; Schwettmann et al. 2019; Zbären et al. 2023). The following regions were included in our DCMs: right early visual areas (visual), right supramarginal gyrus (SMG), right superior parietal lobule (SPL), right dorsal premotor cortex (PMd) overlapping with frontal eye fields (FEF), and right supplementary motor area (SMA). We restricted our ROIs to the right hemisphere to limit model complexity and due to stronger activations of the right hemisphere in the PPI analysis. For each ROI, we first defined a fixed outer sphere with a radius of 16 mm, centred on the MNI coordinates of the group-level right hemisphere peak activations in the PPI analysis (i.e., visual [9–88 -14], SMG [56 − 36 52], SPL [20–70 50], PMd [30 0 52], and SMA [6 − 2 64]). To account for individual differences in functional anatomy, we defined a mobile inner sphere with a radius of 6 mm, centred on subject-specific peak activations from a ‘physical inference > control’ contrast from GLM1.

The peak activations were located within both the outer sphere and a mask of the right hemisphere obtained from the Harvard-Oxford subcortical structural atlas (Desikan et al. 2006). Time-series were extracted from the voxels within the inner sphere that exceeded an uncorrected threshold of *p* < .05. In three participants, one of the five ROIs did not contain any surviving voxels so we lowered their threshold until a peak voxel could be identified (i.e., to *p* < .1 for two participants and *p* < .2 for the third participant), as recommended in Zeidman et al. (2019). We did not exclude these participants as someone with a weak or absent response in one brain region may still provide valuable information about the other regions in the network. Also note that the use of a threshold for time-series extraction is only to remove the noisiest voxels.

##### First-level DCM specification and inversion

**Fig. 2:**
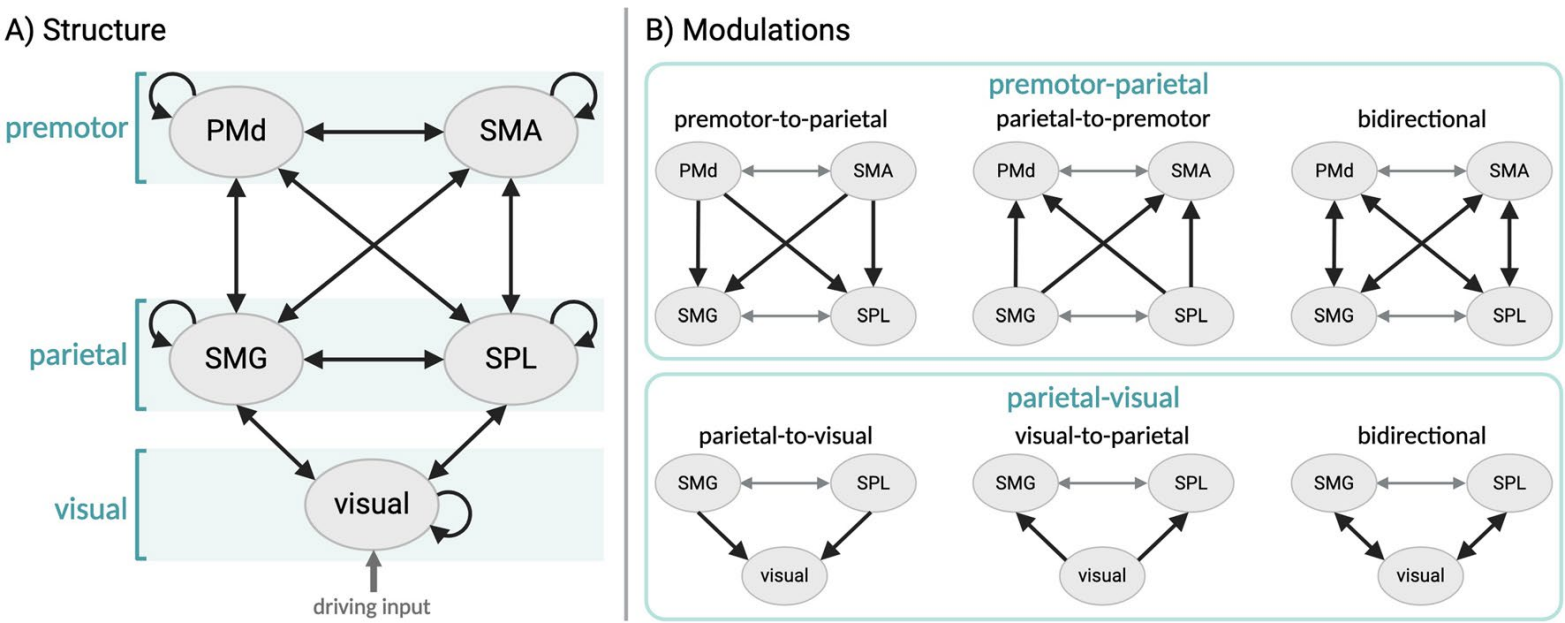
Representation of the model space for DCM. **A**. Endogenous connectivity (i.e., $A$-matrix). **B**. Possible modulatory effects (i.e., $B$-matrix) on premotor-parietal connections (top panel) and parietal-visual connections (bottom panel). Each type of parietal-visual modulation was combined with each type of premotor-parietal modulation, resulting in a total of nine models (i.e., full PEB model and 8 reduced PEB models). Grey arrows represent fixed modulations that are identical across models

In the *A* matrix, we specified reciprocal intrinsic connections between the following pairs of regions: visual and SMG, visual and SPL, SMG and SPL, SMG and PMd, SMG and SMA, SPL and PMd, SPL and SMA, and PMd and SMA (Fig. 2A), in accordance with the anatomical literature (Bakola et al. 2013; Boussaoud et al. 2005; Felleman and Van Essen 1991; Luppino et al. 1993). We did not specify any connection between premotor and early visual regions, as there is no compelling evidence of direct anatomical connections (Felleman and Van Essen 1991). The driving input (i.e., the ‘occlusion’ regressor from GLM2) was specified as entering the network via visual regions as the onset of this phase was marked by a change in the screen colour. The inputs were mean-centred, such that the parameter estimates in matrix *B* represent changes in effective connectivity relative to the average connectivity across conditions (i.e., physical inference and control). In the *B* matrix, we specified a ‘full’ DCM in which all the between-region connections present in the *A* matrix could be modulated by physical inference. The DCM for each subject was then inverted, thereby providing estimates of the connectivity parameters that best explain the data.

##### Second-level analysis using parametric empirical Bayes

The subject-specific connectivity parameter estimates were then taken to the group level, where we used parametric empirical Bayes (PEB; Friston et al. 2015) together with Bayesian Model Reduction (BMR; Friston et al. 2016) to test hypotheses on the group-level connectivity parameters. We collated the previously estimated fully connected model of each subject to estimate a second-level PEB model on the $B$ matrix parameters.

To investigate which connections are most likely to be modulated by physical inference, we defined a set of hypotheses expressed as pre-defined reduced PEB models in which certain connections have been switched off. We specified models in which the following sets of connections could be modulated by physical inference: from visual to parietal (i.e., SMG and SPL) regions, from parietal to visual regions, or both, and from parietal (i.e., SMG and SPL) to premotor (i.e., PMd and SMA) regions, from premotor to parietal regions, or both (Fig. 2B). Each type of modulation between visual and parietal regions (i.e., visual-to-parietal, parietal-to-visual, bidirectional) could be combined with each type of modulation between parietal and premotor regions (i.e., parietal-to-premotor, premotor-to-parietal, bidirectional), resulting in a total of nine models. We allowed reciprocal connections between the premotor (PMd and SMA) and between the parietal (SMG and SPL) regions to always be modulated by physical inference, as there was no compelling reason to assume they would not be. This was done to limit the number of models and because these connections were not of particular interest for the current analysis. Together with the full model, our model space consisted of nine models.

We then tested which of our pre-defined models best explains the commonalities across subjects, by comparing the log-evidence of the full PEB model against reduced ones. Since none of the models could be categorised as a winning one (i.e., probability > 95%, see S1 of the Supplementary Material), we averaged the parameters across models using Bayesian model averaging (BMA; Hoeting et al. 1999). BMA yields weighted averages of parameter estimates, where each parameter estimate is weighted by the posterior probability of the associated model, thereby characterizing the direction and size of task-related changes in connectivity strength (i.e., expressed in the matrix $B$). To determine the statistical significance of the parameter estimates, we set a threshold based on free energy and retained the parameters with a posterior probability of being present versus absent ≥ 0.95. Additionally, to test whether and which modulations of effective connectivity were associated with time estimation performance and/or self-rated vividness (see Sect. 2.3), we performed two stepwise multiple linear regression analyses with the DCM parameters informed by the group used as predictors.

## Results

### Behavioural data

In the physical inference task, the mean absolute fall time errors were of 0.5441 s ± 0.1466, and the self-reported vividness scores had a mean of 5.525 ± 2.2549.

### Psychophysiological interaction analysis

**Fig. 3:**
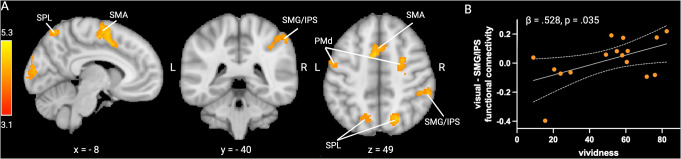
Results of the PPI analysis. **A**. Group random-effects activation map of the PPI analysis, thresholded at Z > 3.1 and FWE-corrected using a cluster significance level of p_FWE_ < 0.05. **B**. Association between parameter estimates of the right SMG/IPS cluster and self-rated vividness. The grey shading represents the 95% confidence interval

We performed a PPI analysis to identify which regions exhibit condition-dependent increases in functional connectivity with early visual areas. Our results revealed increased functional connectivity during physical inference, as compared with the control condition, between early visual areas and a network of frontoparietal regions. This network comprises bilateral dorsal premotor cortex (PMd) with the right hemispheric activation overlapping with frontal eye field (FEF), bilateral supplementary motor area (SMA), bilateral superior parietal lobule (SPL), and right supramarginal gyrus (SMG) with activations extending into the intraparietal sulcus (IPS) (see Fig. 3A and Table S1 of the Supplementary material).

To assess whether functional connectivity changes are relevant for behavioural performance and/or the self-rated vividness of the imagined physical scene, we performed two stepwise multiple linear regressions with the mean parameter estimates of each cluster used as predictors. We found that functional coupling between early visual areas and the SMG significantly predicted self-rated vividness (β = 0.528, *p* = .035) (Fig. 3B), whereas there were no significant linear associations between functional connectivity and time estimation performance.

### Dynamic causal modelling

Bayesian model selection (BMS) revealed that the model that best explains the data, with a probability of 89%, is the one that allows physical inference to modulate top-down parietal-visual and bidirectional parietal-premotor connections, followed by the full model with a probability of 11% (Figure S1). However, since no model’s probability reached 95%, a clear winning model could not be determined. Consequently, Bayesian model averaging (BMA) was used to estimate model parameters.

**Fig. 4:**
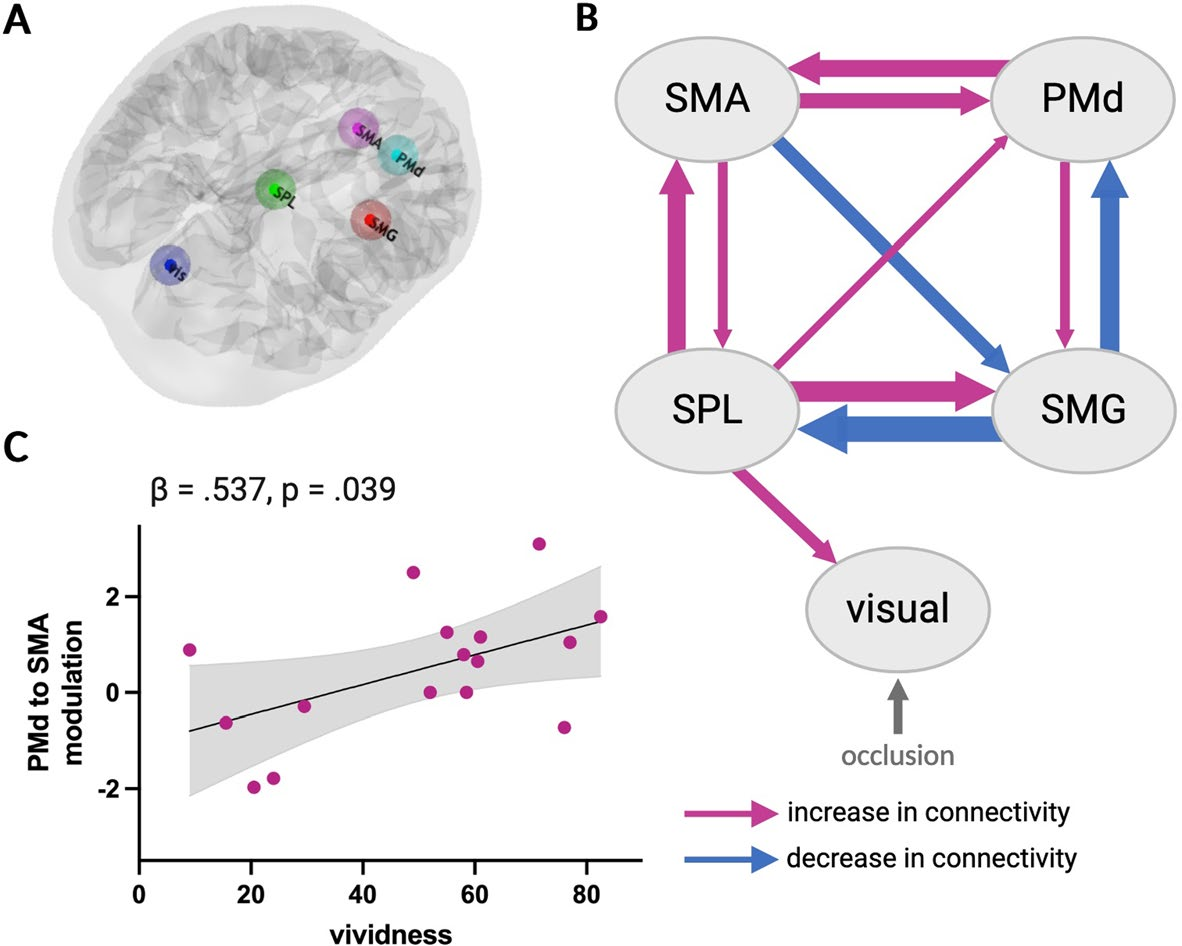
Dynamic causal modelling analysis. **A**. Location of regions of interest in an example subject: dark blue = early visual, green = SPL, red = SMG, pink = SMA, cyan = PMd. **B**. Depiction of the parameter estimates computed using PEB/BMA and exhibiting a probability ≥ 0.95. Pink arrows indicate an increase in connectivity and blue arrows indicate a decrease in connectivity associated with physical inference, relative to the average connectivity across conditions. The thickness of each arrow is proportional to the average size of the corresponding parameter estimate. **C**. Association between the parameter estimates of the PMd to SMA modulation and self-rated vividness. The grey shading represents the 95% confidence interval

The results of our PEB/BMA analysis showed that physical inference was associated with strong decreases in effective connectivity from SMG to SPL and PMd while the strongest increases of effective connectivity originated from SPL and projected to SMG, SMA, and early visual areas (see Fig. 4B and Table S2 of the Supplementary Material). Our results further show bidirectional increases of connectivity between premotor areas (i.e., SMA and PMd). Interestingly, the results did not reveal any significant modulation of connectivity between SMG and early visual areas.

We found an association between increased effective connectivity from PMd to SMA and heightened self-rated vividness (β = 0.537, *p* < .05) (Fig. 4C). There were no significant linear associations between effective connectivity changes and time estimation performance.

## Discussion

The aim of the present study was to examine connectivity patterns among brain regions involved in physical inference when predicting the trajectory of objects falling parabolically and under occlusion. Participants underwent fMRI while performing a task in which they had to infer the trajectory of an occluded ball falling from various heights and with various horizontal velocities, and a visually matched control task. Building on our previous research demonstrating that when solving this task, early visual areas contain representations of the occluded trajectory similar to those activated by observing a ball fall (Zbären et al. 2023), the present study aimed to (i) identify regions that interact with early visual areas during the physical inference task, and (ii) investigate changes in effective connectivity within this network of regions. We found that during physical inference, early visual areas are functionally connected to a set of parietal and premotor regions. Our DCM results further show that physical inference is associated with bidirectional changes in effective connectivity within a parieto-premotor network and increased top-down coupling from the SPL to early visual areas.

### Early visual areas are functionally connected to frontoparietal regions when predicting the trajectory of occluded objects

Our psychophysiological interaction (PPI) analysis revealed that during physical inference, the functional coupling between early visual areas and several frontoparietal regions increases in a condition-dependent manner. Specifically, the supramarginal gyrus (SMG) and intraparietal sulcus (IPS), superior parietal lobule (SPL), dorsal premotor cortex (PMd) including frontal eye field (FEF), and supplementary motor area (SMA) show increased connectivity with early visual areas, compared to a visually matched control task. Note that these results are unlikely to be driven by involuntary eye movements, as demonstrated by our prior control experiment in which participants performed the same task while their eye movements were monitored using eye tracking, revealing no difference in eye positions and saccades between the physical inference and control conditions (for further details, see Zbären et al. 2023). All these frontoparietal regions have been shown to be consistently involved in intuitive physical inference (Fischer et al. 2016; Pramod et al. 2022; Schwettmann et al. 2019), and to contain trajectory-specific information about the occluded ball when solving this task (Zbären et al. 2023). This network of regions overlaps with areas typically involved in visuospatial attention (Corbetta and Shulman 2002; Nobre 2001) and visual imagery (Winlove et al. 2018). Our PPI analysis suggests that this frontoparietal network may contribute to evoking activity in early visual areas even in the absence of external visual input, consistent with previous research demonstrating the dorsal frontoparietal network’s role in the top-down allocation of attention not only towards external sensory stimuli but also towards internal representations (Cona and Scarpazza 2019). During visual imagery, dorsal premotor and parietal areas have been shown to modulate early visual activity (Dentico et al. 2014; Dijkstra et al. 2017; Ishai et al. 2000; Mechelli 2004). Additionally, we found that the strength of the functional coupling between the SMG/IPS cluster and early visual areas is a significant predictor of the subjective vividness of the imagined scene. This finding aligns with previous research showing a positive relation between the subjective vividness of a mental image and the strength of the connection from the intraparietal sulcus to early visual area (Dijkstra et al. 2017).

### Intuitive physical inference modulates effective connectivity within a visuo-fronto-parietal network

Given that functional connectivity cannot offer insights into the direction of information flow between connected areas, we further investigated the network of regions revealed by the PPI analysis in terms of effective connectivity. We built anatomically plausible dynamic causal models (DCM) of these regions and how their interactions may be modulated by physical inference. Bayesian model selection did not reveal a clear winning model as none of the models exhibited a probability greater than 95%. Nevertheless, the DCM with the highest model evidence tentatively suggests that physical inference modulates visual-parietal connections in a top-down fashion and parietal-premotor connections bidirectionally.

Inference on model parameters using Bayesian model averaging revealed that physical inference is associated with bidirectional increases in connectivity between SMA and SPL, and between SMA and PMd. The SMA has been closely linked to imagery, particularly motor imagery (Hétu et al. 2013), but also other modalities such as visual imagery (Palmiero et al. 2009). Interestingly, we found the increase in connectivity from the PMd to the SMA to be predictive of the self-rated vividness of the inferred physical scene. However, it is worth noting that the size of our sample may constrain the generalisability of such brain-behaviour relationships. Additionally, the SMA is involved in temporal processing (Hinton et al. 2004; Macar et al. 1999; Nani et al. 2019). Research has shown that when estimating time-to-contact, humans rely not only on kinematic cues derived from visual inputs (e.g., velocity), but also on temporal information, particularly during occlusion (Battaglini and Ghiani 2021; Chang and Jazayeri 2018). Considering this, it is plausible that the SMA might have been implicated in tracking time during the occluded fall in our physical inference task, thereby contributing to predicting the ball’s landing time.

Our results further suggest that solving the physical inference task is associated with pronounced connectivity changes of the SPL, with an increase in effective connectivity from SPL to SMG/IPS and visual areas. It is likely that our SPL ROI, which was centred around MNI_xyz_= 20, -70, 50, contains the human homologue of non-human primates’ area V6Ad, which has been shown to respond to coherent visual motion stimuli and be involved in spatial trajectory planning, particularly in the context of pointing movements (Sulpizio et al. 2023). While this area has been implicated in various tasks requiring the processing of visual information (including continuously moving stimuli) and trajectory planning (Sulpizio et al. 2020, 2023), our analysis suggests a top-down influence of this area on early visual processing during physical inference. This top-down influence may facilitate the activation of visual representations of the physical scenario, resulting in the formation of perception-like images in the absence of bottom-up visual inputs. Interestingly, V6Ad has been shown to be specifically activated by imagery tasks (Sulpizio et al. 2020). Additionally, the SPL increases connectivity bidirectionally with the SMA and unidirectionally to PMd, indicating that key areas of the dorso-medial parieto-premotor processing stream become increasingly connected. In contrast, the SMG/IPS, which is typically considered to be part of the dorso-lateral processing stream, decreases its connectivity with both SPL and PMd. In summary, our findings put forward the hypothesis that performing the physical inference task is associated with stronger bidirectional effective connectivity within a dorso-medial parieto-premotor processing stream, which seems to drive activity in early visual areas and SMG/IPS in a top-down fashion.

Previous work has found that visual imagery is associated with increased top-down coupling from the intraparietal sulcus to early visual regions (Dijkstra et al. 2017), however, in our task, SPL seems to mediate this potential interaction via increased connectivity to both SMG/IPS and early visual areas. Note that the dorso-medial parieto-premotor pathway is considered to constitute the ‘vision-for-action’ pathway, which has traditionally been identified using sensorimotor tasks that involve trajectory planning, for example during reaching (Greulich et al. 2020). Our task did not require participants to directly translate visuo-spatial information into the spatial control of movement, indicating that even though these pathways are essential for motor actions, they seem to underpin more general aspects of behaviour that consider information about the physical world.

Our results did not reveal changes in effective connectivity between early visual areas and the SMG/IPS. This may be explained by the SMG/IPS cluster containing the anterior portion of the IPS (AIP), which does not receive direct input from early visual areas, and recent work showing that also effective connectivity from AIP to early visual areas is limited, as estimated based on resting-state fMRI (Rolls et al. 2023a, b). We did not find any significant associations between changes in functional or effective connectivity and time-estimation performance, suggesting that subject-specific temporal errors may not primarily emerge from suboptimal brain connectivity between two specific areas. This is not entirely unexpected since time estimation performance is likely to reflect the integration of neural processing within a distributed network across the whole duration of the physical inference task.

## Conclusion

Our study shows that when solving a physical inference task requiring participants to infer projectile motion under occlusion, early visual areas are functionally connected to a set of parietal and premotor regions. Our dynamic causal modelling results suggest that predicting occluded trajectories is associated with changes in bidirectional effective connectivity within a dorso-medial parieto-premotor network, which may drive activity in early visual areas in a top-down fashion. These findings offer new insights into the interaction between early visual and physics-responsive frontoparietal regions during physical inference, shedding new light on the neural mechanisms underlying the ability to make predictions about the physical environment.

## Electronic supplementary material

Below is the link to the electronic supplementary material.

Supplementary Material 1

## Data Availability

Data are openly available on the ETH Library Research Collection with the 10.3929/ethz-b-000578094.
