# Supplementary material for: Inferring occluded projectile motion changes connectivity within a visuo-fronto-parietal network
Source: Brain Struct Funct. 2024 Jun 25;229(7):1605–15. doi: 10.1007/s00429-024-02815-2 (PMC11374914; doi:10.1007/s00429-024-02815-2)
Supplement: Supplementary file 1 — Supplementary Material 1 [file 429_2024_2815_MOESM1_ESM.pdf]

Supplementary information

cluster	# voxels	region	pFWEcorr.	peak coordinate x	y	z	z score
1	1434	Supplementary motor area	0	-2	-2	60	4.99
2	387	Superior parietal lobule	0	20	-70	50	5.27
3	356	V2	0	-4	-94	16	4.23
4	351	Superior parietal lobule	0	-20	-70	60	4.19
5	242	Supramarginal gyrus	0	56	-36	52	4.32
6	189	Premotor cortex/Middle frontal gyrus	0.001	30	0	52	4.79
7	96	Premotor cortex	0.029	-56	-4	44	3.83
8	95	V2	0.03	10	-96	16	4.26

Table S1. Activation clusters and corresponding size, anatomical region, FWE-corrected p-value, peak coordinate in MNI space, and maximum z-score of the functional connectivity analysis map, thresholded at $Z > 3.1$ and FWE-corrected using a cluster significance level of $p < .05$. Reported anatomical labels were determined using the Jülich Histological (Eickhoff et al., 2007) and Harvard-Oxford cortical structural (Desikan et al., 2006) atlases, and correspond to the location of maxima within each cluster.

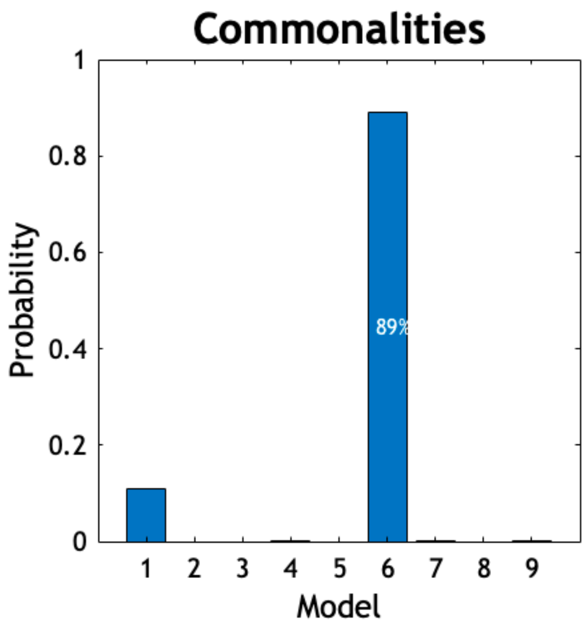

Figure S1. Results of the comparison of the 9 pre-defined PEB models, showing the posterior probability for the commonalities across subjects.

Connection	Effect of physical inference
SPL to visual	0.725
SPL to SMG	1.263
SPL to PMd	0.473
SPL to SMA	1.107
SMG to SPL	-1.558
SMG to PMd	-1.180
PMd to SMG	0.601
PMd to SMA	1.034
SMA to SPL	0.6
SMA to SMG	-0.584
SMA to PMd	0.93

Table S2. Estimated group-level connection strength with units in Hertz (Hz), representing the changes in effective connectivity due to task-related modulatory input, averaged over PEB models. Abbreviations: SPL = superior parietal lobule; SMG = supramarginal gyrus; PMd = dorsal premotor cortex; SMA = supplementary motor areas.
